# Radiofrequency ablation for the treatment of multifocal or large-scale high-grade intraepithelial neoplasia of the esophagus: A safe & effective approach

**DOI:** 10.12669/pjms.40.1.8927

**Published:** 2024

**Authors:** Yang Yang, Liang Liu, Jingyu Zhu

**Affiliations:** 1 Yang Yang, Shandong First Medical University & Shandong Academy of Medical Sciences; Central Hospital Affiliated to Shandong First Medical University, Jinan, Shandong, 250011, China; 2 Liang Liu, Central Hospital Affiliated to Shandong First Medical University, 105 Jiefang Road, Lixia District, Jinan, 250011, Shandong, China; 3 Jingyu Zhu, Central Hospital Affiliated to Shandong First Medical University, 105 Jiefang Road, Lixia District, Jinan, 250011, Shandong, China

**Keywords:** RFA, HGIN, Multifocal, Large-scale, Esophagus

Endoscopic radiofrequency ablation (RFA) is a relatively novel endoscopic therapeutic technique. RFA achieves therapeutic purpose by attaching high-frequency electrode plate to the lesion and releasing radiofrequency wave to burn off the lesion from the mucosa and submucosa. High-grade intraepithelial neoplasia (HGIN) of the esophagus refers to dysplastic proliferation of squamous epithelium in the esophagus, with dysplastic cells accumulating in more than half of the epithelial layer without breaking through the basement membrane. The presence of the HGIN significantly increases the potential risk of evolving into invasive esophageal squamous cell carcinoma (ESCC).^1^ Endoscopic curative methods for the HGIN including endoscopic mucosal resection (EMR), endoscopic submucosal dissection (ESD) and the RFA. It is worth noting that for multifocal or large-scale HGIN, particularly those involving more than two-thirds of circumference of the esophagus, completely lesion resection with endoscopic resection (ER) becomes considerably challenging, with higher rates of intraoperative bleeding and perforation, as well as a higher incidence of postoperative stricture. At the same time, large or multifocal HGIN with the ER requires experienced endoscopists and various endoscopic techniques, meanwhile, long-time endoscopic treatment such as EMR or ESD will inevitably increase the anesthesia-related risks.

RFA is relatively simple and has a short operating time, it can eliminate superficial lesions at depths of 0.5-1mm, thereby completely eradicating HGIN that is confined to the mucosal layer. Therefore, in the endoscopic treatment of large-scale and multifocal HGIN, RFA has the advantages of shorter operative time and less trauma than the EMR and ESD. At the same time, patients have less postoperative pain and shorter duration of discomfort, which is also an advantage of the RFA.^2^ Importantly, our goal in treating HGIN is not only to achieve complete response (CR) but also to maximize patient prognosis improvement and minimize the occurrence of complications. The most common and refractory complication of endoscopic treatment for multifocal and large-scale HGIN is postoperative luminal stenosis.^3^ The mechanism of the RFA significantly reduces the risk of post-esophageal stricture compared to the EMR and ESD, leading to a significant enhancement in patient quality of life.

Currently, RFA has been widely applied in the treatment of Barrett’s esophagus with associated precancerous lesions and chronic erosive gastritis. Additionally, RFA has shown promising prospects in the treatment of mucosal leukoplakia, radiation enteritis and other gastrointestinal disorders. In recent years, our endoscopic center has accumulated a wealth of experience in the treatment of patients with multifocal and large-scale HGIN of the esophagus.

**Fig.1:**
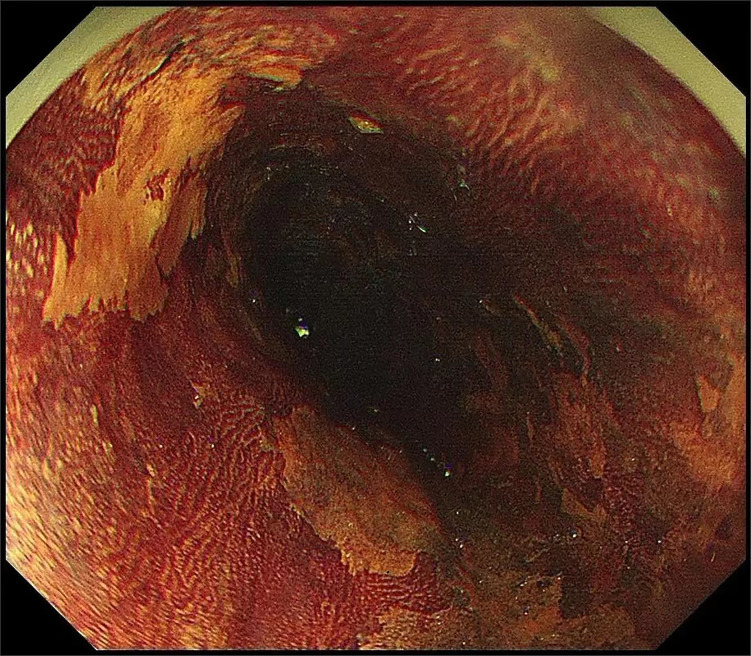
Iodine staining observation of multiple light stained areas. (Biopsy pathology is HGIN or Low-grade intraepithelial neoplasia).

**Fig.2:**
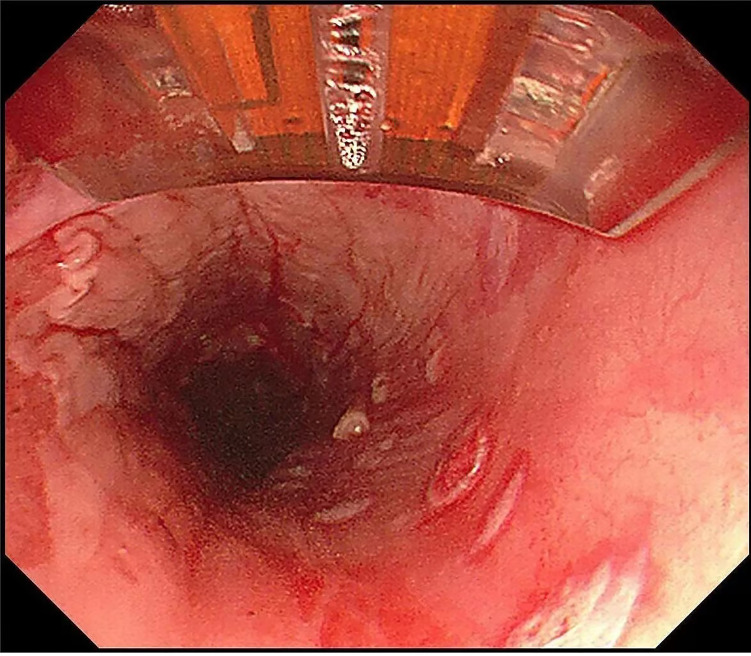
Perform RFA ablation of lesions.

Most notably, it is an urgent need to initiate multicenter randomized controlled trials to obtain more valuable clinical data. In summary, we believe that the RFA is a safe and effective minimally invasive endoscopic treatment method for multifocal or large-scale esophageal high-grade intraepithelial neoplasia.

## Authors Contribution

**YY** conceived, designed and prepared draft of the manuscript.

**LL** is responsible for revising the manuscript and integrity of research.

**JZ** is responsible for collecting and organizing patient information, and has reviewed and finally approved the manuscript.
